# Systemic Immune-Inflammation Index and Clinical Predictors of Atypical PRES in Eclampsia: Higher Blood Pressure and Inflammatory Burden Drive Multi-Regional Involvement

**DOI:** 10.3390/biomedicines14040862

**Published:** 2026-04-09

**Authors:** Mehmet İncebıyık, Adalet Göçmen

**Affiliations:** 1 Department of Obstetrics and Gynecology, Faculty of Medicine, Harran University, Şanlıurfa 63050, Türkiye; 2 Department of Neurology, Faculty of Medicine, Harran University, Şanlıurfa 63050, Türkiye; dradaletyavuzgocmen@gmail.com

**Keywords:** eclampsia, Posterior Reversible Encephalopathy Syndrome, atypical PRES, neuroimaging burden, severe hypertension

## Abstract

**Objective**: To identify clinical and neuroimaging predictors of atypical Posterior Reversible Encephalopathy Syndrome (PRES) in eclampsia and evaluate the role of multi-regional cerebral involvement (neuroimaging burden). **Methods**: This retrospective cohort study included 266 patients with eclampsia and radiologically confirmed PRES (2018–2025). Patients were classified as typical (*n* = 234, 88.0%) or atypical (*n* = 32, 12.0%). A two-stage multivariable logistic regression was performed to identify independent predictors, sequentially incorporating clinical and neuroimaging variables. **Results**: Peak systolic blood pressure was significantly higher in atypical vs. typical groups (191.6 ± 20.4 vs. 172.4 ± 18.5 mmHg, *p* < 0.001). Furthermore, atypical cases exhibited a significantly higher systemic inflammatory burden, characterized by markedly elevated Systemic Immune-Inflammation Index (SII) and CRP levels (*p* < 0.001). Atypical cases exhibited a markedly greater neuroimaging burden, with a higher mean number of involved brain regions (4.4 ± 1.2 vs. 2.1 ± 0.6, *p* < 0.001). In Model 1 (clinical variables only), systolic blood pressure was a strong predictor of atypicality (OR: 1.24 per 10 mmHg increase, 95% CI: 1.12–1.38, *p* < 0.001). After incorporating neuroimaging features in Model 2, the total number of involved brain regions emerged as the strongest independent predictor (OR: 2.08, 95% CI: 1.52–2.85, *p* < 0.001), while the independent effect of blood pressure was attenuated. **Conclusions**: Atypical PRES in eclampsia reflects extensive, high-burden cerebral vasogenic edema rather than a distinct radiological subtype. While hypertension initiates the process, the total regional burden determines the atypical signature. This burden-focused perspective improves risk stratification and diagnostic vigilance in high-risk obstetrics.

## 1. Introduction

Posterior Reversible Encephalopathy Syndrome (PRES) is a complex clinicoradiological entity defined by a constellation of neurological symptoms, ranging from headache and visual disturbances to generalized seizures and coma, coupled with characteristic vasogenic edema on neuroimaging [1]. While PRES is associated with diverse clinical triggers such as immunosuppression, renal failure, and systemic lupus erythematosus, eclampsia remains its most dramatic and life-threatening manifestation [2]. In the obstetric population, PRES is not merely a neurological complication but a systemic reflection of severe endothelial dysfunction and a catastrophic failure of cerebral autoregulation, often occurring within the unique hemodynamic milieu of pregnancy [1,3].

The classic “canonical” radiological presentation of PRES is characterized by relatively symmetrical vasogenic edema, primarily localized within the posterior circulation territories, specifically the parietal and occipital lobes [1,4]. This posterior predilection has historically been attributed to the sparse sympathetic innervation of the vertebrobasilar system compared to the anterior circulation, which renders the posterior regions vulnerable to sudden elevations in systemic blood pressure and subsequent breakdown of the blood–brain barrier [5]. However, as the utilization of high-resolution neuroimaging has expanded, it has become increasingly evident that eclampsia-related PRES frequently defies this traditional “posterior” nomenclature. Atypical manifestations, involving the frontal lobes, deep gray matter (thalamus and basal ganglia), brainstem, and cerebellum, are reported with increasing frequency, yet their clinical significance and underlying triggers remain a subject of intense academic debate [6].

A critical gap persists in the current literature regarding why certain eclamptic patients develop these atypical distribution patterns. One prevailing hypothesis suggests that atypical PRES is a distinct clinical phenotype driven by specific inflammatory markers or systemic complications like HELLP syndrome [7]. Alternatively, others propose that atypicality is simply a marker of disease severity, a “spillover” effect where extreme hypertensive peaks overcome the autoregulatory capacity of the entire cerebral vasculature, not just the vulnerable posterior territories [1,3,8]. Our cohort data supports this severity-driven model, as atypical presentations were associated with significantly higher systolic and diastolic blood pressures compared to typical patterns.

The distinction between typical and atypical patterns is not a mere radiological exercise; it carries profound implications for clinical management and maternal-fetal outcomes. Atypical presentations, particularly those involving deep gray matter or the brainstem, can mimic acute stroke or central venous thrombosis, potentially leading to diagnostic delays or inappropriate interventions in the peripartum period [9]. Moreover, understanding whether atypicality is driven by absolute blood pressure peaks or by the cumulative burden of multi-regional cerebral involvement is essential for refining neuro-prognostication [10].

In this study, we leveraged a large, well-characterized cohort of 266 eclampsia patients to systematically investigate the clinical and radiological predictors of atypical PRES. By utilizing a robust multivariable approach, we aimed to dissect whether atypicality is an independent clinical trait or a consequence of the extent of regional vasogenic edema. We hypothesized that while hypertension serves as the primary trigger, the ultimate emergence of an atypical topography in eclampsia is fundamentally determined by the “total regional burden”—the number of brain territories involved—rather than isolated clinical markers. This study aims to provide a comprehensive statistical and visual map of atypical PRES, offering new insights into the radiological architecture of this critical obstetric-neurological syndrome.

## 2. Materials and Methods

### 2.1. Study Design and Ethical Approval

This retrospective, cross-sectional study was conducted at a tertiary care referral university hospital specializing in high-risk obstetrics. The study protocol was approved by the Institutional Clinical Research Ethics Committee (Session No: 20; Protocol No: HRÜ/25.20.60). Due to the retrospective nature of the data analysis, the requirement for informed consent was waived by the ethics committee. All procedures were performed in accordance with the ethical standards of the Declaration of Helsinki.

### 2.2. Patient Selection and Inclusion Criteria

A systematic screening of the institutional electronic medical database was performed to identify all pregnant or postpartum patients diagnosed with eclampsia between 2018 and 2025. The initial cohort consisted of 312 patients presenting with new-onset seizures and/or neurological deficits.

Patients were included based on the following criteria: clinical diagnosis of eclampsia; availability of high-quality neuroimaging (MRI or CT) performed within 48 h of neurological symptom onset, and radiological confirmation of vasogenic edema consistent with Posterior Reversible Encephalopathy Syndrome (PRES).

**Figure 1:**
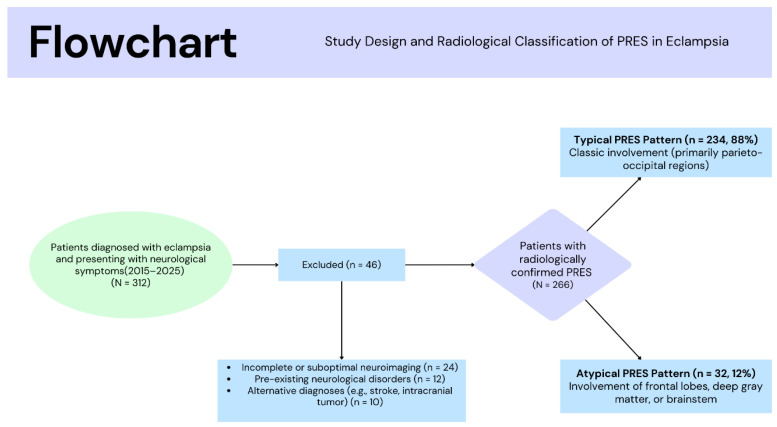
Flowchart of the Study Population and Radiological Classification.

Exclusion criteria were pre-existing neurological disorders such as epilepsy or structural brain lesions (*n* = 12), alternative diagnoses such as ischemic stroke, intracranial hemorrhage, or tumor (*n* = 10), and suboptimal image quality or incomplete clinical data (*n* = 24). The final study population consisted of 266 patients (Figure 1).

### 2.3. Neuroimaging Acquisition and Assessment

While CT was utilized as an adjunct for acute evaluation in 45.9% of cases (*n* = 122), MRI served as the definitive imaging modality for all 266 patients (100%). Standard MRI protocols included T1-, T2-, FLAIR-, and Diffusion-Weighted Imaging (DWI) sequences.

Two senior neuroradiologists (with 10 and 15 years of experience, respectively), blinded to clinical and laboratory severity markers, independently reviewed all images. To ensure reliability and minimize subjectivity, any discrepancies in regional involvement or classification were resolved by a third senior radiologist through a consensus-based approach to ensure diagnostic accuracy. Inter-observer agreement for the topographical classification of PRES was substantial, with a Cohen’s kappa coefficient of 0.84.

The assessment focused on the following parameters:

Topographical Pattern Classification: Patients were categorized into Typical (predominantly parieto-occipital) and Atypical patterns. Following rigorous anatomical relabeling, atypicality was defined by the presence of edema in non-canonical regions, including the frontal lobes, deep gray matter (thalamus, basal ganglia), cerebellum, or brainstem, regardless of the presence of posterior involvement. Specifically, atypicality did not imply the absence of typical parieto-occipital involvement; rather, it represented an extensive multi-regional ‘overflow’ pattern where atypical regions were involved in addition to, or as a progression of, classical territories.

Neuroimaging Burden (Regional Count): While automated volumetric analysis was not utilized, the extent of the syndrome was quantified using the “Total Region Count,” defined as the sum of involved brain territories (frontal, parietal, occipital, temporal, deep gray matter, cerebellum, and brainstem). This metric served as a proxy for the global burden of cerebral vasogenic edema and was assessed using a standardized scoring system previously validated in similar neuroimaging studies.

### 2.4. Clinical and Laboratory Data Collection

Comprehensive clinical data were extracted from medical records, including maternal age, gestational age at delivery, parity, and peak blood pressure (systolic and diastolic) recorded during the eclamptic event. Laboratory parameters, including platelet counts, liver enzymes (AST, ALT), and creatinine levels, were recorded. To minimize information bias and reflect the acute phase of the syndrome, all laboratory samples and blood pressure measurements were obtained within the first 6 h of hospital admission following the eclamptic seizure. The presence of HELLP syndrome was defined according to the Tennessee Classification criteria.

### 2.5. Statistical Analysis

Statistical analyses were performed using SPSS version 26.0 (IBM Corp., Armonk, NY, USA) and R software (version 4.3.1; R Foundation for Statistical Computing, Vienna, Austria). Continuous variables were expressed as mean ± standard deviation (SD) or median with interquartile range (IQR), and categorical variables were presented as frequencies and percentages. Group comparisons (Typical vs. Atypical) were performed using the Mann–Whitney U test, Chi-square test, or Fisher’s exact test.

Variables for the multivariable logistic regression models were selected based on their clinical relevance and statistical significance (*p* < 0.10) in univariate analyses. To prevent multicollinearity, highly correlated inflammatory markers were evaluated using variance inflation factors (VIF), and the most representative indices (such as SII) were retained for the final models.

To identify independent predictors of the atypical PRES pattern, a two-stage multivariable logistic regression analysis was performed. Model 1 evaluated clinical parameters (maternal age, peak systolic blood pressure, and HELLP syndrome). Model 2 incorporated neuroimaging features (total region count, deep gray involvement, and time to imaging) to determine the independent effect of regional burden while adjusting for clinical severity.

Receiver Operating Characteristic (ROC) curve analysis was performed to assess the model’s predictive performance, including the calculation of the Area Under the Curve (AUC) and overall model accuracy. A sensitivity analysis was performed to evaluate the stability of the multivariable models, which confirmed that the identified predictors remained consistent across different clinical subgroups. Missing data for primary variables were less than 5%; therefore, a complete case analysis was performed without the need for formal imputation methods. A *p*-value < 0.05 was considered statistically significant.

## 3. Results

### 3.1. Study Population and Baseline Characteristics

A total of 312 patients with a clinical diagnosis of eclampsia were initially screened. After applying the predefined inclusion and exclusion criteria, 266 patients were included in the final analysis (Figure 1). The majority of patients demonstrated a typical radiological PRES pattern (*n* = 234, 88.0%), whereas 32 patients (12.0%) exhibited an atypical PRES pattern.

The median maternal age of the study population was 29.0 years (interquartile range [IQR]: 25.0–32.0), and 69.5% of the patients were multiparous. Intravenous magnesium sulfate (MgSO_4_) was administered as first-line anticonvulsant therapy in 99.2% of cases.

### 3.2. Comparison of Typical and Atypical PRES Patterns

Clinical, laboratory, and obstetric characteristics of patients with typical and atypical PRES patterns are summarized in Table 1. There were no statistically significant differences between the groups in terms of maternal age (28.9 ± 5.6 vs. 27.5 ± 4.8 years, *p* = 0.196) or gestational age at delivery (34.5 ± 3.3 vs. 34.7 ± 4.2 weeks, *p* = 0.387).

In contrast, patients with an atypical PRES pattern presented with significantly higher blood pressure values. Mean systolic blood pressure was markedly higher in the atypical group compared with the typical group (191.6 ± 20.4 vs. 172.4 ± 18.5 mmHg, *p* < 0.001). Similarly, diastolic blood pressure was significantly elevated in atypical cases (118.5 ± 14.2 vs. 108.2 ± 12.4 mmHg, *p* < 0.001).

The prevalence of HELLP syndrome was also significantly greater among patients with atypical PRES (25.0% vs. 8.9%, *p* = 0.048, Table 1). Maternal and neonatal outcomes, including intensive care unit (ICU) length of stay and 5-min APGAR scores, did not differ significantly between the two radiological patterns (all *p* > 0.05, Table 1).

### 3.3. Neuroimaging Findings and Regional Distribution

**Figure 2:**
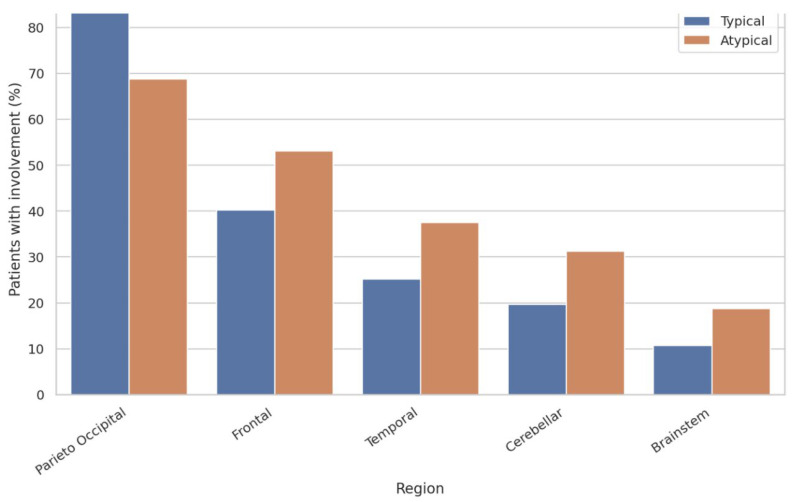
Comparison of Regional Brain Involvement Between Typical and Atypical PRES Patterns.

Distinct differences in the distribution of vasogenic edema were observed between typical and atypical PRES patterns (Figure 2, Table 1). Although parieto-occipital involvement was common in both groups, atypical PRES was associated with a significantly higher frequency of frontal (87.5% vs. 45.3%), temporal (62.5% vs. 15.4%), and cerebellar (53.1% vs. 12.8%) involvement (*p* < 0.001 for all comparisons).

**Figure 3:**
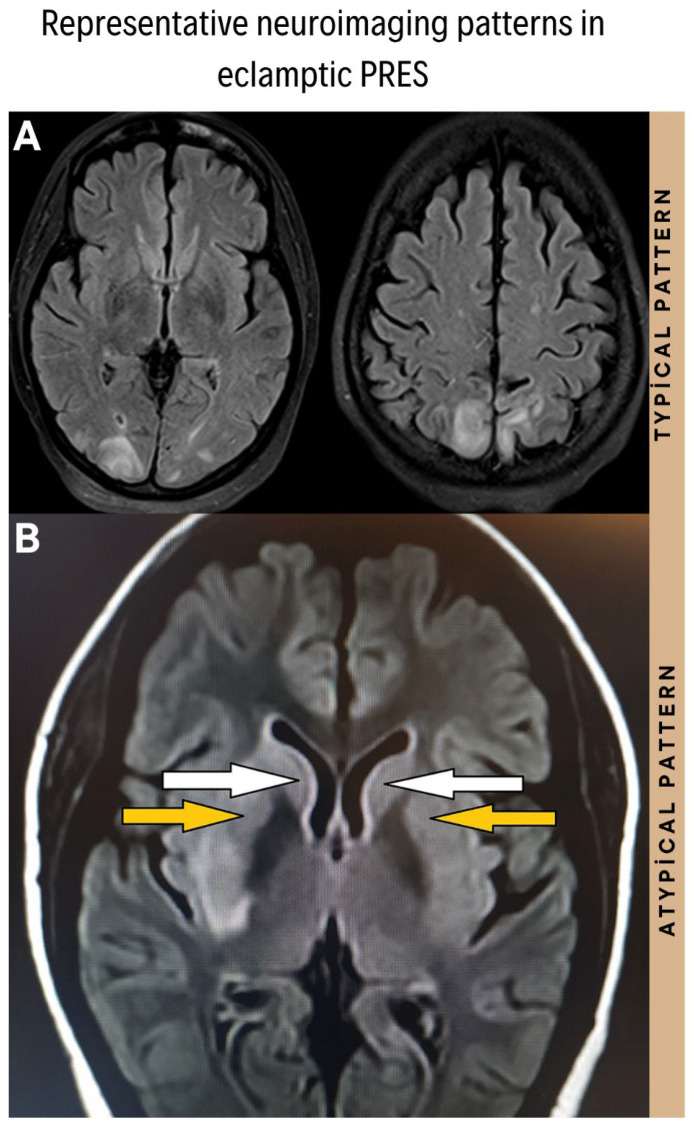
Representative neuroimaging patterns (Axial FLAIR MRI) in patients with eclampsia. (**A**) Typical PRES Pattern (Upper Panel): Axial FLAIR image reveals characteristic symmetrical hyperintense signals (vasogenic edema) primarily localized in the parieto-occipital lobes. (**B**) Atypical PRES Pattern (Lower Panel): Axial FLAIR image demonstrates extensive involvement of non-canonical regions, including the deep gray matter (thalamus and basal ganglia), representing a high-burden phenotype. White arrows indicate hyperintensities in the head of the caudate nucleus bilaterally, and yellow arrows indicate hyperintensities in the putamen region bilaterally.

Deep gray matter involvement was identified in more than half of the atypical cases (56.3%), compared with 23.1% in the typical PRES group (*p* < 0.001). Representative examples of these distinct typical and atypical distribution patterns are illustrated in Figure 3. Importantly, the overall regional burden, defined as the total number of involved brain regions, was substantially greater in patients with atypical PRES (4.4 ± 1.2 regions) than in those with typical PRES (2.1 ± 0.6 regions, *p* < 0.001).

### 3.4. Predictors of Atypical PRES Pattern

The results of the two-stage multivariable logistic regression analysis are presented in Table 2. In Model 1, which included clinical variables only, peak systolic blood pressure emerged as a significant predictor of atypical PRES. Each 10 mmHg increase in systolic blood pressure was associated with a 24% increase in the odds of an atypical radiological pattern (OR: 1.24, 95% CI: 1.12–1.38, *p* < 0.001, Table 2).

In Model 2, incorporating neuroimaging parameters, the regional burden was identified as the strongest independent predictor of atypical PRES (OR: 2.08, 95% CI: 1.52–2.85, *p* < 0.001, Table 2). Notably, inclusion of the regional burden attenuated the independent effect of systolic blood pressure, suggesting that the association between hypertension and atypical PRES may be mediated through the extent of cerebral involvement.

**Figure 4:**
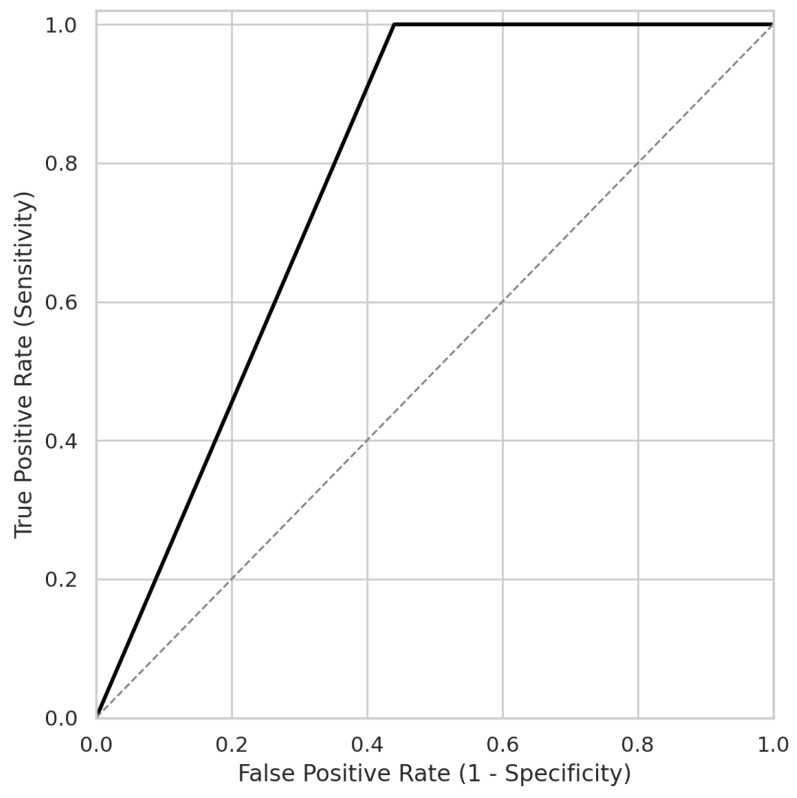
Receiver Operating Characteristic (ROC) Curve of the Multivariable Model for Predicting Atypical PRES. The model, incorporating peak systolic blood pressure and regional burden, demonstrates strong discriminative performance with an Area Under the Curve (AUC) of 0.89 and an overall diagnostic accuracy of 88.4%. The dotted line represents the reference line (diagonal line of no discrimination).

Although renal function markers (including creatinine and proteinuria) showed a trend toward higher values in the atypical group, they were not significantly associated with the atypical PRES pattern in the multivariable analysis after adjusting for blood pressure and inflammatory indices. ROC curve analysis further confirmed the model’s performance, with an AUC of 0.89 and an overall accuracy of 88.4% (Figure 4).

### 3.5. Immunological Profile and Inflammatory Burden in Typical vs. Atypical PRES

The systemic inflammatory and immunological profile differed significantly between the groups (Table 3). Patients in the atypical PRES group exhibited a markedly higher inflammatory burden compared to the typical group. Specifically, the mean Systemic Immune-Inflammation Index (SII) was significantly elevated in atypical cases (1965.8 ± 450.6 vs. 1240.5 ± 310.2, *p* < 0.001). Similarly, the Neutrophil-to-Lymphocyte Ratio (NLR) and CRP levels were substantially higher in the atypical group (*p* < 0.001 for both). Additionally, the atypical group showed evidence of more severe endothelial and metabolic stress, characterized by higher LDH levels (512.4 ± 124.8 vs. 345.2 ± 88.6 U/L, *p* = 0.004) and significantly lower albumin concentrations (2.6 ± 0.5 vs. 3.1 ± 0.4 g/dL, *p* = 0.018).

## 4. Discussion

In this large-scale multivariable analysis of 266 patients with eclampsia, we investigated the determinants of atypical Posterior Reversible Encephalopathy Syndrome (PRES) patterns. The principal finding of our study is the clear dissociation between crude clinical associations and adjusted multivariable interpretations. Although atypical PRES was strongly associated with extreme hypertensive peaks in univariate analyses, our multivariable models demonstrate that atypicality is significantly associated with the extent of cerebral involvement rather than isolated indicators of clinical severity. Specifically, atypical PRES appears to be an independent function of the multi-regional burden of vasogenic edema.

These findings suggest that, in eclampsia, atypical PRES represents a disseminated manifestation of widespread endothelial dysfunction rather than a distinct radiological phenotype triggered solely by excessive blood pressure elevation. Our results therefore support a pathophysiological model in which the distribution of cerebral involvement reflects the global severity of autoregulatory failure rather than regional susceptibility alone.

### 4.1. Hypertension Threshold and the Sympathetic Innervation Hypothesis

The classical posterior predominance of PRES has traditionally been attributed to the relative paucity of sympathetic innervation in the vertebrobasilar circulation compared with the anterior cerebral circulation [1,3,11]. This anatomical vulnerability is thought to lower the threshold for autoregulatory failure within the parieto-occipital regions [11]. However, the 12% prevalence of atypical PRES observed in our cohort adds to growing evidence that the eclamptic brain frequently deviates from this traditional posterior distribution pattern [12].

Previous studies have suggested that involvement of the frontal lobes, temporal lobes, or brainstem reflects a more severe PRES phenotype precipitated by hypertensive breakthroughs [13]. Although our univariate analyses confirmed significantly higher blood pressure levels in patients with atypical patterns, multivariable modeling provided a more nuanced interpretation. After adjustment for the total number of involved brain regions, the independent predictive effect of peak systolic blood pressure was no longer statistically significant (OR: 0.91, *p* = 0.182). This finding indicates that hypertension likely acts as the initiating insult disrupting blood–brain barrier integrity, whereas atypical imaging patterns reflect a generalized and sustained failure of cerebrovascular autoregulation unique to the eclamptic state rather than a purely pressure-driven focal phenomenon [14].

### 4.2. The “Overflow Theory” of Multi-Regional Involvement

The most compelling observation in our analysis was the strong and independent association between the extent of cerebral involvement and atypical PRES. Each additional involved brain region was associated with more than a twofold increase in the likelihood of atypicality (OR: 2.08, 95% CI: 1.52–2.85). Based on this observation, we propose an “Overflow Theory” of eclamptic PRES. According to this framework, as the underlying pathophysiological insult characterized by acute endothelial dysfunction, systemic inflammation, and impaired autoregulation intensifies, vasogenic edema progressively extends beyond canonical posterior territories into non-classical brain regions [5].

Supporting this theory, our study provides novel insights into the role of systemic inflammation in the pathogenesis of atypical involvement. We found that patients in the atypical PRES cohort exhibited a significantly higher inflammatory burden, characterized by markedly elevated Systemic Immune-Inflammation Index (SII), NLR, and CRP levels compared to typical cases (*p* < 0.001) [15]. We propose that this heightened immunological state acts synergistically with hypertension to exacerbate blood–brain barrier (BBB) disruption. Specifically, the combination of high inflammatory mediators and low albumin levels observed in our atypical group likely lowers the threshold for vasogenic edema, facilitating its extension into non-classical brain areas. These results indicate that the immunological profile of the patient is a critical determinant of the radiological phenotype, supporting the view that atypical PRES is a more severe, immunologically active variant of the disease [4].

Notably, although deep gray matter involvement was highly prevalent among atypical cases (56.3%), it did not retain independent predictive significance after adjustment for overall regional burden. This suggests that thalamic or basal ganglia involvement rarely represents an isolated anatomical event but instead serves as a surrogate marker of widespread cerebral dissemination and advanced disease severity [9,16]. Collectively, these findings support a conceptual shift from a location-centered to a burden-centered understanding of atypical PRES.

### 4.3. Clinical Implications and Obstetric Management

From an obstetric perspective, the significantly higher prevalence of HELLP syndrome among patients with atypical PRES underscores the close relationship between systemic multi-organ dysfunction and extensive cerebral involvement. Nevertheless, reliance on systemic clinical markers alone appears insufficient for predicting atypical neuroimaging patterns [17].

Atypical PRES presents a substantial diagnostic challenge, as involvement of the frontal lobes, brainstem, or deep gray nuclei may mimic ischemic stroke, intracranial hemorrhage, or cerebral venous sinus thrombosis. This diagnostic overlap may lead to delayed or inappropriate management during the critical peripartum period [3,16,18]. Our findings emphasize that deviations from the classical posterior pattern should not be regarded merely as radiological variants but rather as imaging markers of extensive multi-regional cerebral disease. Accordingly, aggressive and timely management of eclampsia should be pursued irrespective of the apparent severity of initial clinical parameters [3,9,19].

The observed atypical PRES incidence of 12.0% in our cohort is higher than some reports in the general obstetric population. However, this likely reflects a selection bias inherent to our institution’s role as a major tertiary referral center. Since the most severe and complex eclampsia cases from the region are primarily managed at our hospital, this potentially concentrates higher-burden phenotypes, providing a unique opportunity to study these atypical presentations in a large-scale analysis. The multivariable model developed in this study showed high discriminative power (AUC: 0.89), suggesting that the combination of severe hypertensive peaks and the extent of vasogenic edema (regional burden) can reliably identify patients at risk for atypical PRES patterns. This high accuracy (88.4%) underscores the clinical utility of integrating neuroimaging severity with clinical parameters.

## 5. Limitations

Several limitations should be acknowledged. First, the retrospective design and our institution’s role as a tertiary referral center may have introduced selection bias toward more complex cases, potentially explaining the higher prevalence of atypical PRES compared to the general obstetric population. Second, the timing of laboratory tests and neuroimaging was not strictly synchronized with eclampsia onset, which may result in temporal variations in blood pressure levels and systemic inflammation markers. Third, the assessment of neuroimaging involvement relied on expert consensus rather than automated volumetric techniques, which may have limited the precision of burden quantification. Fourth, although atypical PRES was characterized by greater radiological and inflammatory burden, the lack of significant differences in ultimate maternal and neonatal outcomes (such as ICU stay or APGAR scores) may reflect the high level of specialized care provided at our center rather than a lack of biological severity. Finally, residual confounding related to the rate of blood pressure escalation or the precise timing of magnesium sulfate administration cannot be fully excluded.

## 6. Conclusions

In conclusion, atypical PRES in eclampsia primarily reflects extensive multi-regional cerebral involvement rather than isolated clinical severity markers. While severe hypertension initiates the pathological cascade, the total regional burden of vasogenic edema constitutes the defining neuroimaging signature of atypicality. This burden-focused perspective has important implications for risk stratification, diagnostic vigilance, and neuro-prognostication in high-risk obstetric populations. Recognizing that atypicality is a marker of widespread cerebral dissemination should guide clinicians toward more aggressive management in eclamptic patients exhibiting non-canonical imaging patterns.

## Figures and Tables

**Table 1:** Demographic, Clinical, Neuroimaging, and Outcome Characteristics According to PRES Pattern.

Characteristic	Overall (*n* = 266)	Typical PRES (*n* = 234)	Atypical PRES (*n* = 32)	OR (95% CI)	*p*-Value
Demographics					
Maternal age (years)	29.0 (25.0–32.0)	28.9 ± 5.6	27.5 ± 4.8	0.95 (0.88–1.03)	0.196
Gestational age (weeks)	34.6 (32.3–36.9)	34.5 ± 3.3	34.7 ± 4.2	1.01 (0.91–1.13)	0.387
Parity ≥ 1, *n* (%)	185 (69.5%)	164 (70.1%)	21 (65.6%)		0.305
Clinical Findings					
Peak systolic BP (mmHg)	179.0 (165.2–195.0)	172.4 ± 18.5	191.6 ± 20.4	1.24 (1.12–1.38)	<0.001 *
Peak diastolic BP (mmHg)	112.0 (103.0–121.0)	108.2 ± 12.4	118.5 ± 14.2	1.16 (1.09–1.24)	<0.001 *
HELLP syndrome, *n* (%)	29 (10.9%)	21 (8.9%)	8 (25.0%)	3.38 (1.36–8.40)	0.048 *
MgSO_4_ administered, *n* (%)	264 (99.2%)	232 (99.1%)	32 (100%)		1.000
Laboratory Parameters					
Platelets (×10^3^/µL)	165 (122–209)	165.0 (122.0–208.8)	163.5 (120.8–217.0)		0.915
AST (U/L)	66 (44–98)	65.0 (43.2–98.0)	75.5 (46.2–94.2)		0.734
ALT (U/L)	58 (38–86)	58.5 (36.0–88.0)	59.0 (46.5–80.2)		0.703
Neuroimaging Features					
Time to imaging (hours)	9.1 (5.9–14.2)	8.8 (5.6–13.8)	11.2 (7.4–16.5)		0.342
Deep gray involvement, *n* (%)	72 (27.1%)	54 (23.1%)	18 (56.3%)	4.28 (2.04–8.99)	<0.001 *
Regions involved, count	3 (2–3)	2.1 ± 0.6	4.4 ± 1.2	2.08 (1.52–2.85)	<0.001 *
Maternal & Neonatal Outcomes					
Emergent C-section, *n* (%)	253 (95.1%)	223 (95.3%)	30 (93.8%)		0.660
ICU length of stay (days)	4.0 (1.0–15.0)	4.0 (1.0–15.0)	4.0 (2.0–15.0)	1.02 (0.92–1.14)	0.691
Mechanical ventilation, *n* (%)	60 (22.6%)	52 (22.2%)	8 (25.0%)		0.821
APGAR score (5th min)	8 (7–9)	8.0 (1.0–10.0)	8.0 (5.0–10.0)	0.98 (0.85–1.12)	0.948

Values are presented as mean ± SD or median (IQR), unless otherwise indicated. Abbreviations: BP, blood pressure; CI, confidence interval; HELLP, hemolysis, elevated liver enzymes, low platelet count; ICU, intensive care unit; MgSO_4_, magnesium sulfate; OR, odds ratio. * Statistically significant (*p* < 0.05).

**Table 2:** Multivariable Logistic Regression Analysis for Atypical PRES.

Predictor	Adjusted OR	95% CI	*p*-Value
Model 1: Clinical Factors			
Maternal age (per year)	0.96	0.89–1.03	0.254
Peak systolic BP (per 10 mmHg)	1.24	1.12–1.38	<0.001 *
HELLP syndrome (yes)	1.84	0.72–4.68	0.201
Model 2: Neuroimaging Features			
Regions involved (per region)	2.08	1.52–2.85	<0.001 *
Deep gray involvement (yes)	1.62	0.82–3.21	0.165
Time to imaging (per hour)	0.98	0.94–1.02	0.342

Note: OR, odds ratio; CI, confidence interval; BP, blood pressure. Adjusted odds ratios were derived from multivariable logistic regression models. Asterisks (*) indicate statistical significance (*p* < 0.05).

**Table 3:** Comparison of Systemic Immune-Inflammation Markers Between Typical and Atypical PRES Groups.

Parameters	Typical PRES (*n* = 234)	Atypical PRES (*n* = 32)	OR (95% CI)	*p*-Value
NLR (Neutrophil/Lymphocyte Ratio)	6.42 ± 2.15	9.85 ± 3.42	1.32 (1.15–1.52)	<0.001
PLR (Platelet/Lymphocyte Ratio)	142.6 ± 45.2	188.4 ± 62.8	1.01 (1.00–1.02)	0.011
SII (Systemic Immune-Inflammation Index)	1240.5 ± 310.2	1965.8 ± 450.6	1.14 (1.08–1.21)	<0.001
CRP (mg/L)	14.8 ± 8.4	28.6 ± 12.5	1.12 (1.06–1.19)	<0.001
LDH (U/L)	345.2 ± 88.6	512.4 ± 124.8	1.01 (1.00–1.02)	0.004

Note: Data are presented as mean ± standard deviation (SD). Abbreviations: NLR, neutrophil-to-lymphocyte ratio; PLR, platelet-to-lymphocyte ratio; SII, systemic immune-inflammation index; CRP, C-reactive protein; LDH, lactate dehydrogenase. OR (95% CI) values represent univariate associations. Statistically significant (*p* < 0.05).

## Data Availability

The datasets generated and/or analyzed during the current study are not publicly available due to institutional policies and patient privacy protections. De-identified data may be made available upon reasonable request to the corresponding author, subject to approval by the institutional review boards of the participating hospitals.

## References

[1] Fischer M., Schmutzhard E. (2017). Posterior reversible encephalopathy syndrome. J. Neurol..

[2] Triplett J., Kutlubaev M., Kermode A., Hardy T. (2022). Posterior reversible encephalopathy syndrome (PRES): Diagnosis and management. Pract. Neurol..

[3] Hinduja A. (2020). Posterior Reversible Encephalopathy Syndrome: Clinical Features and Outcome. Front. Neurol..

[4] Ollivier M., Bertrand A., Clarençon F., Gerber S., Deltour S., Domont F., Trunet S., Dormont D., Leclercq D. (2017). Neuroimaging features in posterior reversible encephalopathy syndrome: A pictorial review. J. Neurol. Sci..

[5] Anderson R., Patel V., Sheikh-Bahaei N., Liu C., Rajamohan A., Shiroishi M., Kim P., Go J., Lerner A., Acharya J. (2020). Posterior Reversible Encephalopathy Syndrome (PRES): Pathophysiology and Neuro-Imaging. Front. Neurol..

[6] Wen Y., Yang B., Huang Q., Liu Y. (2017). Posterior reversible encephalopathy syndrome in pregnancy: A retrospective series of 36 patients from mainland China. Ir. J. Med. Sci..

[7] Álvarez-Pabón Y., Beltrán-Avendaño M., Di Lizio-Miele K. (2017). Síndrome de encefalopatía posterior reversible, eclampsia y síndrome de hellp. Rev. Chil. De Obstet. Y Ginecol..

[8] Bartynski W. (2008). Posterior Reversible Encephalopathy Syndrome, Part 2: Controversies Surrounding Pathophysiology of Vasogenic Edema. Am. J. Neuroradiol..

[9] Saad A., Chaudhari R., Wintermark M. (2019). Imaging of Atypical and Complicated Posterior Reversible Encephalopathy Syndrome. Front. Neurol..

[10] Srichawla B., Garcia-Dominguez M., Silver B. (2025). The Central Variant of Posterior Reversible Encephalopathy Syndrome: A Systematic Review and Meta-Analysis. Neurol. Int..

[11] Gewirtz A., Gao V., Parauda S., Robbins M. (2021). Posterior Reversible Encephalopathy Syndrome. Curr. Pain Headache Rep..

[12] Brewer J., Owens M., Wallace K., Reeves A., Morris R., Khan M., LaMarca B., Martin J. (2013). Posterior reversible encephalopathy syndrome in 46 of 47 patients with eclampsia. Am. J. Obstet. Gynecol..

[13] Chiang W., Chen P., Chen Y., Chen M. (2019). Atypical posterior reversible encephalopathy syndrome in a noncompliant hemodialysis patient: Case report and literature review. Hemodial. Int..

[14] Cipolla M. (2007). Cerebrovascular function in pregnancy and eclampsia. Hypertension.

[15] Zhang X., Chen Y., Fan Y., Gao D., Zhang Z. (2025). NLR (neutrophil to lymphocyte ratio), PLR (platelet to lymphocyte ratio), and SII (systemic immune-inflammation index) reflect disease activity and renal remission in patients with lupus nephritis. Front. Immunol..

[16] Mert G., Kaya Ö., Shahveranova A., Bıçakçı Y. (2025). Comparison of magnetic resonance imaging, clinical, and laboratory findings in atypical and typical posterior reversible encephalopathy syndrome. Cukurova Med. J..

[17] Incognito G., Genovese F., Incognito D., Volpicelli A., Chieppa P., Saponaro C., Palumbo M. (2022). Postpartum HELLP syndrome associated with posterior reversible encephalopathy syndrome: A case report and literature review. Ital. J. Gynaecol. Obstet..

[18] Vennapusa J., Godavarthy P., Garikipaty S., Kulkarni M., Chippa S. (2025). A (PRES)sing Matter: Two Cases of Posterior Reversible Encephalopathy Syndrome (PRES) in Pregnancy and Postpartum. Cureus.

[19] Del Poggio A., Narcisi L., Mapelli R., Lombardi L., Falini A., Anzalone N. (2025). Clinico-radiological correlations in posterior reversible encephalopathy syndrome: Toward a better understanding of its heterogeneous manifestations. Neuroradiology.

